# DESCRIPTIVE EPIDEMIOLOGY ON THE TRENDS OF SUICIDAL ATTEMPTS AMONG OLDER ADULTS IN KOREA FROM 2007–2020

**DOI:** 10.1093/geroni/igad104.2501

**Published:** 2023-12-21

**Authors:** Seoyoon Lee, Hyung Eun Shin, SeungCheor Lee, In-Hwan Oh

**Affiliations:** Yonsei University, Seoul, Republic of Korea; Kyung Hee University, Seoul, Republic of Korea; Kyung Hee University, Seoul, Republic of Korea; Kyung Hee University, Seoul, Republic of Korea

## Abstract

When a suicide attempt results in the act of suicide, personal and social losses increase to a degree that is difficult to quantify. It is urgent to evaluate the status of suicide attempts in Korea accurately. This study is to examine a 14-year time series of older adults who have engaged in suicidal behavior or attempted suicide. We used claim data from Health Insurance Review and Assessment Service. A total of 3,440 individuals with a diagnosis code, 768 individuals with a primary diagnosis, and 2,672 individuals with a secondary diagnosis, were included in the analysis. Suicide attempts were defined by intentional self-harm(X60-X84), harmful biological control agent(X87), and personal history of self-harm(Z91.5). To analyze the frequency of suicide attempts by age, a chi-squared test was conducted. Among those over 60 years with a primary diagnosis of suicide attempt, personal history of self-harm was the most common, accounting for 92.4%, followed by intentional self-harm with a rate of 7.5%. No subjects had a primary diagnosis as code of X87. A similar trend was observed for those under the age of 60. In the case of those with a secondary diagnosis of suicide attempt, personal history of self-harm was the highest at 93.5%. Subsequently, intentional self-harm accounted for about 6.4%, and each code was evenly distributed except for alcohol addiction and exposure(X65) in 2007 and X87 in 2020. Conclusively, this study reported the trend of suicide attempts in Korea, which can provide the basis for preparing effective short- and long-term health policies.

